# The association between glucose 6-phosphate dehydrogenase (G6PD) deficiency and attention deficit/hyperactivity disorder (ADHD)

**DOI:** 10.1192/j.eurpsy.2024.200

**Published:** 2024-08-27

**Authors:** B. Krone, J. Newcorn, I. Manor, E. Merzon

**Affiliations:** ^1^Psychiatry, Icahn School of Medicine at Mount Sinai, New York, United States; ^2^Psychiatry, ADHD Unit, Geha MHC; ^3^Lehumit Health Services, Tel Aviv, Israel

## Abstract

**Introduction:**

Glucose-6-phosphate dehydrogenase (G6PD) deficiency is an X-linked genetic enzymopathy that impacts 4.9% of the population, with greater prevalence among Mediterranean, East Asian, and African populations. G6PD deficiency results in levels of nicotinamide-adenine dinucleotide phosphate (NADPH) and glutathione (GSH) that are insufficient for maintaining the balance of oxidation-reduction in the body. This results in elevated production of reactive oxygen species (ROS), oxidative stress on proteins and lipids, damage to DNA, and potential activation of chemokine and cytokine pathways by astrocytes and microglia. We propose that these direct and indirect effects of G6PD deficiency are associated with development of ADHD.

**Objectives:**

This study investigated the association between G6PD deficiency and Attention Deficit/Hyperactivity Disorder (ADHD).

**Methods:**

The study involved 7,473 G6PD-deficient patients and 29,892 matched case-controls (selected at a 1:4 ratio) from a cohort of 1,031,354 within the Leumit Health Services database. Clinical characteristics were analyzed using Fisher’s Exact Tests for categorical variables and Mann-Whitney U tests for continuous variables.

**Results:**

The average age of patients was 29.2 ± 22.3 years, with 68.7% being male. The mean follow-up duration was 14.3 ± 6.2 years. Individuals with G6PD deficiency showed a significant 16% higher risk of being diagnosed with ADHD (Odds Ratio (OR) = 1.16 [95% CI, 1.08-1.25], p < 0.001) on follow up. Furthermore, G6PD deficiency was associated with a 30% greater likelihood of seeking care from adult neurologists (OR = 1.30 [95% CI, 1.22-1.38], p < 0.001) and a 12% higher probability of consulting adult psychiatrists (OR = 1.12 [95% CI, 1.01-1.24], p = 0.048). The use of stimulant medications among G6PD deficient individuals was 17% higher for methylphenidate class drugs (OR = 1.17 [95% CI, 1.08, 1.27], p < 0.001), and use of amphetamines elevated by 16% (OR = 1.16 [95% CI, 1.03, 1.37], p = 0.047).

**Conclusions:**

This study establishes a significant association between G6PD deficiency and an increased risk of ADHD diagnoses. These findings suggest potential opportunities for the development of culturally sensitive interventions.

**Disclosure of Interest:**

B. Krone Consultant of: HIPPO T&C, Signant Health, J. Newcorn: None Declared, I. Manor: None Declared, E. Merzon: None Declared

